# Rapid nanopore sequencing and predictive susceptibility testing of positive blood cultures from intensive care patients with sepsis

**DOI:** 10.1128/spectrum.03065-23

**Published:** 2024-01-09

**Authors:** Patrick N. A. Harris, Michelle J. Bauer, Lukas Lüftinger, Stephan Beisken, Brian M. Forde, Ross Balch, Menino Cotta, Luregn Schlapbach, Sainath Raman, Kiran Shekar, Peter Kruger, Jeff Lipman, Seweryn Bialasiewicz, Lachlan Coin, Jason A. Roberts, David L. Paterson, Adam D. Irwin

**Affiliations:** 1 UQ Centre for Clinical Research, Faculty of Medicine, University of Queensland, Brisbane, Australia; 2 Central Microbiology, Pathology Queensland, Royal Brisbane and Women’s Hospital, Brisbane, Australia; 3 Herston Infectious Disease Institute, Royal Brisbane and Women’s Hospital Campus, Brisbane, Australia; 4 Ares Genetics GmbH, Carlbergergasse, Vienna, Austria; 5 University Children’s Hospital Zurich, University of Zurich, Zurich, Switzerland; 6 Child Health Research Centre, The University of Queensland, Brisbane, Queensland, Australia; 7 Paediatric Intensive Care Unit, Queensland Children’s Hospital, South Brisbane, Australia; 8 Adult Intensive Care Services, The Prince Charles Hospital, Brisbane, Queensland, Australia; 9 Faculty of Medicine, University of Queensland, Brisbane, Australia; 10 Intensive Care Unit, Princess Alexandra Hospital, Woolloongabba, Queensland, Australia; 11 Department of Anaesthesiology and Critical Care, The University of Queensland, St Lucia, Queensland, Australia; 12 Intensive Care Unit, Royal Brisbane and Women’s Hospital, Brisbane, Australia; 13 Division of Anaesthesiology Critical Care Emergency and Pain Medicine, Nîmes University Hospital, University of Montpellier, Nîmes, France; 14 Jamieson Trauma Institute, Royal Brisbane and Women’s Hospital, Brisbane, Australia; 15 Australian Centre for Ecogenomics, School of Chemistry and Molecular Biosciences, Faculty of Science, University of Queensland, Brisbane, Australia; 16 Department of Microbiology and Immunology, The Peter Doherty Institute for Infection and Immunity, University of Melbourne, Melbourne, Victoria, Australia; 17 Departments of Pharmacy and Intensive Care Medicine, Royal Brisbane and Women’s Hospital, Brisbane, Australia; 18 ADVANCE-ID, Saw Swee School of Public Health, National University of Singapore, Singapore, Singapore; 19 Infection Management and Prevention Service, Queensland Children’s Hospital, Brisbane, Queensland, Australia; JMI Laboratories, North Liberty, Lowa, USA

**Keywords:** long-read whole-genome sequencing, bloodstream infection, nanopore

## Abstract

We aimed to evaluate the performance of Oxford Nanopore Technologies (ONT) sequencing from positive blood culture (BC) broths for bacterial identification and antimicrobial susceptibility prediction. Patients with suspected sepsis in four intensive care units were prospectively enrolled. Human-depleted DNA was extracted from positive BC broths and sequenced using ONT (MinION). Species abundance was estimated using Kraken2, and a cloud-based system (AREScloud) provided *in silico* predictive antimicrobial susceptibility testing (AST) from assembled contigs. Results were compared to conventional identification and phenotypic AST. Species-level agreement between conventional methods and AST predicted from sequencing was 94.2% (49/52), increasing to 100% in monomicrobial infections. In 262 high-quality AREScloud AST predictions across 24 samples, categorical agreement (CA) was 89.3%, with major error (ME) and very major error (VME) rates of 10.5% and 12.1%, respectively. Over 90% CA was achieved for some taxa (e.g., *Staphylococcus aureus*) but was suboptimal for *Pseudomonas aeruginosa*. In 470 AST predictions across 42 samples, with both high quality and exploratory-only predictions, overall CA, ME, and VME rates were 87.7%, 8.3%, and 28.4%. VME rates were inflated by false susceptibility calls in a small number of species/antibiotic combinations with few representative resistant isolates. Time to reporting from sequencing could be achieved within 8–16 h from BC positivity. Direct sequencing from positive BC broths is feasible and can provide accurate predictive AST for some species. ONT-based approaches may be faster but significant improvements in accuracy are required before it can be considered for clinical use.

**IMPORTANCE:**

Sepsis and bloodstream infections carry a high risk of morbidity and mortality. Rapid identification and susceptibility prediction of causative pathogens, using Nanopore sequencing direct from blood cultures, may offer clinical benefit. We assessed this approach in comparison to conventional phenotypic methods and determined the accuracy of species identification and susceptibility prediction from genomic data. While this workflow holds promise, and performed well for some common bacterial species, improvements in sequencing accuracy and more robust predictive algorithms across a diverse range of organisms are required before this can be considered for clinical use. However, results could be achieved in timeframes that are faster than conventional phenotypic methods.

## INTRODUCTION

Sepsis is a major cause of morbidity and mortality. Rapid pathogen identification and antimicrobial susceptibility phenotyping is critical to selection of appropriate treatment and ensuring optimal patient outcomes (1, 2). Current pathogen identification and culture-based antimicrobial susceptibility testing (AST) can take up to 3 days, or longer. Consequently, rapid molecular detection and gene profiling methodologies are needed (2–4), especially in an era of an increasing prevalence of antimicrobial resistance.

We have previously demonstrated the application of Illumina-based sequencing from positive blood culture broths (5). This approach showed reasonable performance in both species-level identification and predictive AST from genomic data, but there were few advantages over conventional methods in terms of turn-around times to clinical reporting. In this study, we aimed to evaluate the use of Oxford Nanopore Technologies (ONT) sequencing, using a similar approach and an established DNA extraction method, to determine whether reductions in turn-around times can be achieved without sacrificing diagnostic performance. We compared a sequencing-based predictive AST tool to conventional culture-based methods in order to determine whether this approach could be applicable in a diagnostic laboratory and achieve acceptable performance characteristics.

## MATERIALS AND METHODS

This was a sub-study of the DIRECT program: a prospective, observational multicentre study of children and adults presenting to the intensive care unit (ICU) with clinical features of sepsis (6). Patients were screened for enrolment in four ICUs (three adult, one paediatric) in Brisbane, Australia (Royal Brisbane and Women’s Hospital, The Prince Charles Hospital, Princess Alexandra Hospital, and Queensland Children’s Hospital). Patients who met inclusion criteria and none of the exclusion criteria were eligible for enrolment (Table 1).

**TABLE 1:** Inclusion and exclusion criteria

Inclusion criteria	Exclusion criteria
a) Age > 1 month	a) Inability to gain informed consent
b) Admitted to ICU	b) Neonates (<1 month age)
c) Decision to treat for suspected sepsis^*b*^	c) Imminent death likely
d) Blood cultures collected within 12 h	d) Palliative care intent
e) Commenced on IV antibiotics within 24 h, or a change in antibiotics initiated within 24 h for a new episode of infection	e) On renal replacement therapy^*a*^ f) Use of extra-corporeal membrane oxygenation (ECMO)^*a*^

^*a*^ Necessary as the main study included therapeutic drug monitoring and dose optimisation.

^*b*^ Defined as suspected/proven infection with evidence of end organ dysfunction.

### Sampling

This study included patients with positive blood cultures and presence of bacteria confirmed by Gram stain and microscopy. Samples with non-bacterial species (e.g., *Candida*) were excluded from further analysis (as the analysis pipelines are optimised for prokaryotic organisms). Samples with likely contamination (e.g., mixed coagulase-staphylococci), which were not worked up further by the clinical lab for identification or susceptibility testing, were also not analysed further. For some samples, susceptibility testing was not routinely performed on cultured isolates (e.g., anaerobes); hence, predictive AST was not assessed.

**Fig 1:**
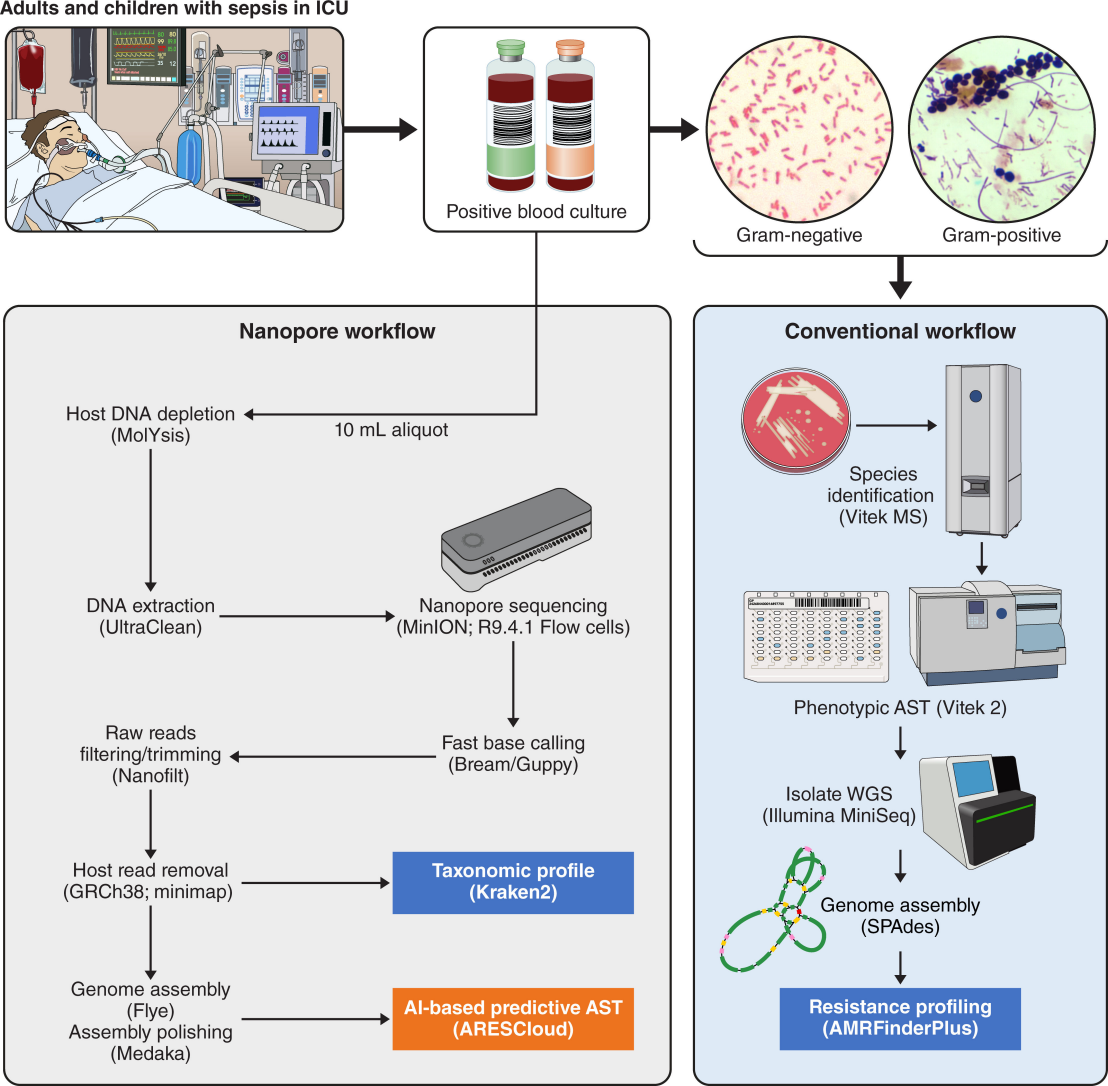
Workflow for Nanopore sequencing and conventional analysis of positive blood cultures.

Blood culture bottles (FA plus, FN plus, and paediatric PF plus bottles; bioMérieux) were removed from BACT/Alert Virtuo System once flagged positive with microbial growth on Gram stain. A de-identified 10 mL aliquot of the positive blood culture broth was processed by a blinded researcher in a separate research lab (The University of Queensland, Centre for Clinical Research), located on the same campus, within 1.5 h (Fig. 1). An uninoculated blood culture broth was also sampled to determine the extent of background DNA contamination. Methods for DNA extraction have been detailed previously (5). In brief, host genomic DNA (gDNA) was depleted using the MolYsis Complete and MolYsis Basic kits (Molzym, Germany) 0.2 mL and 1 mL protocols, respectively, according to manufacturer’s instructions, (with minor modifications), then centrifuged at 10,000*g* for 30 s, and the supernatant removed (5). The microbial pellet then underwent further gDNA extraction using UltraClean kits. Samples were extracted for genomic DNA upon receipt and the remaining sample frozen at −80°C, and if required, thawed to room temperature from frozen.

DNA quality and purity checks were undertaken using the QUBIT fluorometer (Life Technologies), NanoDrop 2000 Spectrophotometer (Thermo Scientific), and Agilent TapeStation 4150 using Genomic DNA ScreenTape and Reagents. For comparison, cultured isolates from the positive BC broths were also retrieved from the clinical laboratory for whole genome sequencing using Illumina (Fig. 1).

### Sequencing

Libraries were prepared using the Rapid Barcoding Sequencing or Nanopore Genomic DNA Ligation kit with Native barcoding according to manufacturer’s instructions (Oxford Nanopore Technologies). Libraries were loaded into the R9.4.1 flow cell and run on the MinION MK1B device. After flow cell quality checks, all sequencing utilized the flow cell priming kit (EXP-FLP002) and sequencing commenced at −180 mV; voltage drift was accounted for where the flow cell went through a wash protocol. The sequencing runs were monitored with MinKNOW version 21.05.25 core 4.3.12. Fast basecalling model was applied with Bream version 6.2.6 and Guppy version 5.0.16 (version 3.2.9 used prior to February 2021). Libraries were run for 72 h but produced adequate data for further analysis within 6–12 h in the majority of samples. Final run QC analysis was undertaken with PycoQC prior to bioinformatic analysis.

In addition, pure colonies of bacteria isolated from positive blood cultures were also sequenced, using DNA extracted by QIAGEN DNeasy Ultraclean Microbial Kit, with quantification by Life Technologies QUBIT fluorometer and library preparation with Illumina ILMN DNA LP tagmentation. Library size was determined by Agilent TapeStation 4150 using D1000 High Sensitivity kit. Sequencing was performed on the Illumina MiniSeq platform with the 300 cycle output reagent kit (Fig. 1).

### Metagenome assemblies, taxonomic profiling, and in-silico WGS-AST

Assembly and binning for predictive AST was performed using a previously published workflow (7). Raw reads were trimmed and quality filtered using Nanofilt 2.8 (8) and mapped against the GRCh38 genome using minimap 2.24 (9) to remove host reads. Taxonomy was assigned using Kraken2 (10). Assignment was done for the whole assembly from all input reads and for metagenomic bins using input reads mapping to the individual metagenomic bin with minimap 2.24. Where possible given input sequencing depth, retained reads were assembled with flye 2.9 (11) and parameters "--nano-raw --meta -- iterations 3." Genome assemblies were polished with Oxford Nanopore Medaka 1.6.1 and parameters "-m r941_min_fast_g303" (12, 13). Binning of assembled metagenomes into metagenomic bins was performed with MaxBin 2.2.7 (14) and MetaBAT 2.15 (13). Bins were unified using DASTool 1.1.5 (15). Resulting bins were post-processed to improve retainment of AMR marker genes from high quality unbinned contigs, as previously described (7). Genome assembly was assessed using checkm2 (16), which uses lineage specific sets of marker genes to estimate completeness and contamination of microbial genomes. Taxonomic profiles were visualized using Krona plots (https://fordegenomics.github.io/direct). Completeness and duplication of bins was assessed with BUSCO (17) and QUAST (18). For each sample, no more than one resulting bin had genome quality metrics compatible with downstream AST prediction. Downstream analysis was, thus, performed on genome assemblies to reduce loss of AMR information in the binning process. Genome assemblies were uploaded to the AREScloud web application, release 2022–10 (Ares Genetics GmbH, Vienna, AT) for genomic prediction of antimicrobial susceptibility. The platform used stacked classification machine learning (ML) predictive AST models trained on ARESdb (19), combined with rule-based resistance prediction via ResFinder 4 (20) to provide species-specific susceptibility/resistance (S/R) predictions. If no high-quality ML models were available in AREScloud for certain taxa, non-specific ResFinder 4 calls based only on generalized presence of antibiotic resistance genes were used but were flagged as being lower confidence predictions. In addition, we compared resistance gene profiling derived from ONT sequencing from BC broths to pure cultured isolates sequenced using Illumina as a reference. Where paired sequence data from both samples were available, *in silico* resistance gene profiles were determined by screening the draft assembled genomes against the NCBI resistance gene database using AMRFinderPlus (version 3.10.24) (21) with default parameters (90% sequence identity and 90% sequence coverage) and compared for concordance in the presence/absence of AMR genes across the sample types.

AST predictions for a total of 25 antibiotic compounds were generated, where appropriate and relevant for that species. True negatives (TN) were defined as data points where both the reference method (phenotypic AST) and the test method (AREScloud) returned a susceptible result; true positives (TP) where both methods returned a resistant result; false positives (FP) where the reference method returned a susceptible and the tested method returned a resistant result; false negatives (FN), where the reference method returned a resistant and the tested method returned a susceptible result. Very major error (VME) and major error (ME) rates were defined following CLSI M52 guidelines (22) as the fraction of cases identified as resistant by the reference method which were identified as susceptible by the tested method [FN/(FN + TP)], and the fraction of cases identified as susceptible by the reference method which were identified as resistant by the tested method [FP/(FP + TN)], respectively. Categorical agreement (CA) between results of WGS-AST and conventional AST were calculated [CA = (TN + TP)/(TN + FP + FN + TP)] for antimicrobial-organism combinations.

### Conventional species identification and AST

All genomics-based species identification and AST results were compared to conventional phenotypic methods validated for clinical use at Pathology Queensland. Species identification was performed using MALDI-TOF (Vitek MS, bioMérieux) on pure cultured isolates, with AST performed by Vitek 2 automated broth microdilution (N-246 AST cards; bioMérieux), using EUCAST clinical breakpoints applicable at the time (23). For certain species (e.g., *Streptococcus pyogenes*), AST was undertaken using disk diffusion according to EUCAST methods (24), or by Etest (bioMérieux) where appropriate (e.g., penicillin for *Streptococcus pneumoniae*). For some species where EUCAST breakpoints were not available (e.g., *Aeromonas* spp.), CLSI breakpoints were applied. Conventional phenotypic testing reported for clinical use by the diagnostic laboratory was considered the reference standard against which genomic results were compared.

## RESULTS

### Blood culture microorganisms

**Fig 2:**
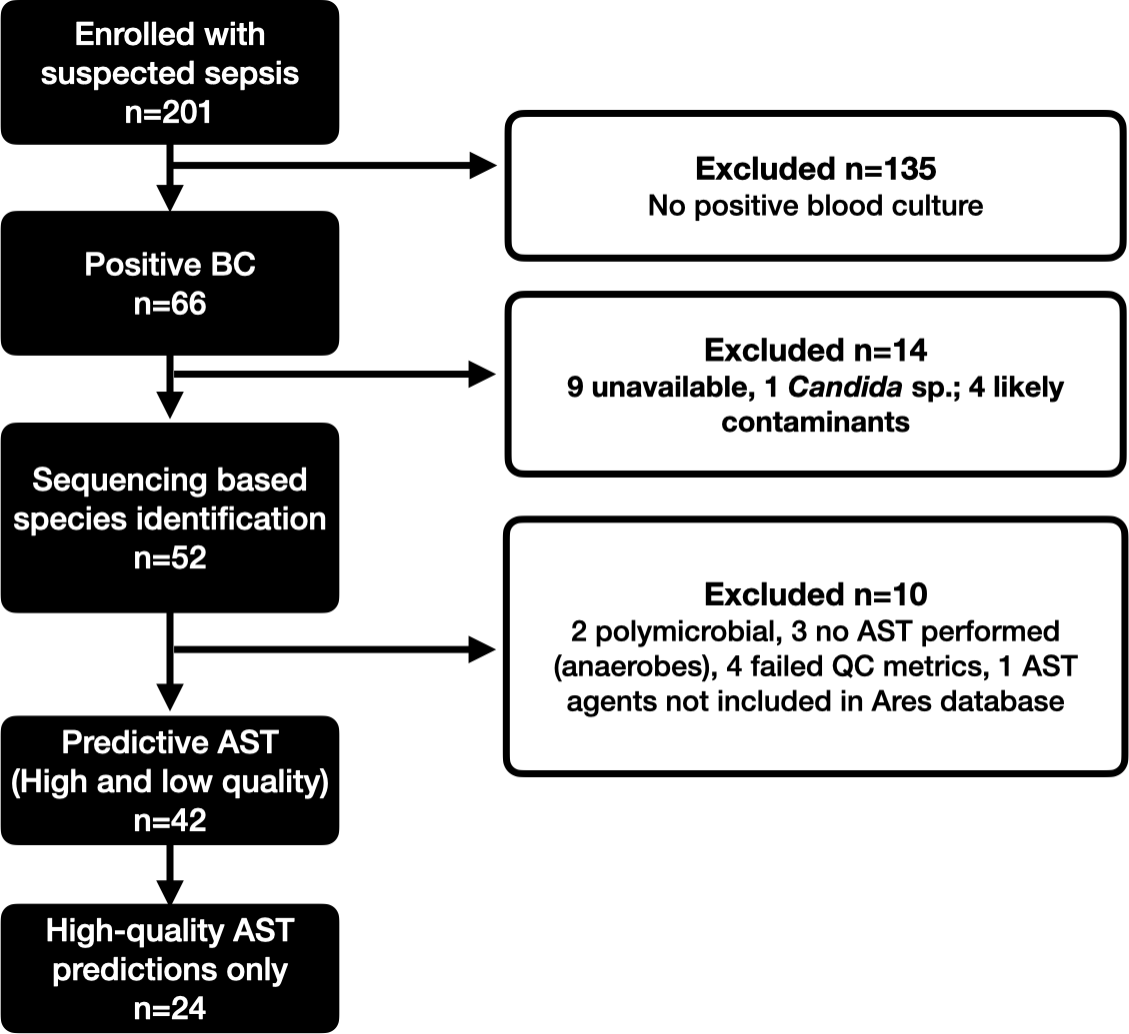
Flow diagram for sample inclusion. BC, blood culture; AST, antimicrobial susceptibility testing; QC, quality control.

A total of 66 positive blood culture samples, from 201 enrolled patients, demonstrated bacterial growth, from which 52 were included for further sequencing analysis, with exclusions reflecting non-bacterial growth (e.g., *Candida* sp.), missing samples or likely contaminants (e.g., mixed coagulase-negative staphylococci) that were not worked up further by the clinical laboratory (Fig. 2; Table S1). Samples included 27 gram-positive and 23 gram-negative bacterial species, with 2 samples showing polymicrobial growth (Table 2).

**TABLE 2:** Species identification by conventional methods

Species	*N*
Gram-positive (*N* = 27)	
*Staphylococcus aureus*	10
*Staphylococcus epidermidis*	7
*Streptococcus pneumoniae*	2
*Streptococcus pyogenes*	1
*Group C Streptococcus*	1
*Staphylococcus capitis*	1
*Staphylococcus lugdunensis*	1
*Staphylococcus haemolyticus*	1
*Enterococcus faecalis*	1
*Clostridium perfringens*	1
*Eggerthella lenta*	1
Gram-negative (*N* = 23)	
*Escherichia coli*	7
*Enterobacter cloacae* complex	4
*Klebsiella (Enterobacter) aerogenes*	1
*Pseudomonas aeruginosa*	3
*Klebsiella pneumoniae*	3
*Haemophilus influenzae*	1
*Stenotrophomonas maltophilia*	1
*Aeromonas* sp.	1
*Prevotella intermedia*	1
*Ochrobactrum anthropi*	1
Polymicrobial (*N* = 2)	
*Escherichia coli + Enterobacter cloacae*	1
*Proteus mirabilis + Staphylococcus hominis*	1
Total	52

### Taxonomy identification from sequenced samples and *in silico* predictive AST

Samples were run either on a single flow cell or multiplexed with up to 12 samples per flow cell, with a median sequencing yield per flow cell of 2.5 Gbp for multiplexed samples and 1.5 Gbp for single samples. Taxonomic identification of sequenced samples yielded excellent agreement to species level compared to conventional methods (49/52, 94.2%); for monomicrobial samples agreement was 98% (49/50). In two samples, genus-level agreement was obtained when compared to Vitek MS, but sequencing provided a more accurate identification; the pipeline identified species belonging to *Enterobacter cloacae* complex (*E. hormaechei* for sample 9420-58 and *E. asburiae* for sample 9420-32) in two samples reported as *E. cloacae* by phenotypic methods. The correct species level identity resulting from sequencing of both samples was confirmed by Illumina-based sequencing of the cultured isolates. For one polymicrobial sample, the secondary pathogen (*E. cloacae*) reported by phenotypic methods was not apparent in the Kraken2 report of input reads, where only one of the cultured pathogens (*E. coli*) was identified. For another polymicrobial sample, the presence of *Staphylococcus hominis* was identified by phenotypic methods alongside *Proteus mirabilis*, but only ~15% of bacterial reads in the sample could be matched to genus *Staphylococcus* and were insufficient for predictive AST. For one sample (9421-30), no identification was achieved due to inadequate input data; with only 145 very short reads (mean read length 191), thus no further processing was possible. Sequencing from an uninoculated blood culture broth revealed a very low number of reads (*n* = 28) mapping to bacterial genomes (e.g., *E. coli*), compared to a mean number of reads of 271411 mapping to bacterial taxa for positive culture broths included in the sequencing analysis.

A total of 470 phenotypic AST results with matched predictive AST calls were analyzed. As conventional AST was not routinely performed in all samples (e.g., likely contaminants, anaerobic organisms), predictive AST was only compared where phenotypic results were available. In addition, two polymicrobial samples were excluded. For an additional six samples, neither exploratory nor high quality AST predictions, were available (four samples had insufficient reads for assembly and in two samples, antibiotics reported by phenotypic methods, were not available in the AREScloud database). As such, a total of 42 samples had both predictive AST and phenotypic AST calls compared, and for 24 samples, high-quality AREScloud predictions were available.

Overall CA was 89.3% for 262 AST results across 24 samples for which quality sequencing-based AST predictions were available, including seven common BSI organisms (*Staphylococcus aureus, Enterococcus faecalis, Escherichia coli, Klebsiella aerogenes, Klebsiella pneumoniae, Streptococcus pneumoniae,* and *Pseudomonas aeruginosa*), but with 12.1% VMEs (mainly seen in *E. coli* with tobramycin and trimethoprim-sulfamethoxazole, driven by a single *E. coli* sample with poor assembly metrics) and 10.5% MEs (mainly seen in *P. aeruginosa* against cefepime, ciprofloxacin, gentamicin, and meropenem; *K. aerogenes* against ceftazidime and ceftriaxone; *E. coli* against amikacin, cephazolin, ampicillin, and cefoxitin; and *K. pneumoniae* against cephazolin and ciprofloxacin) (Tables 3 and 4; Table S2).

**TABLE 3:** Performance of predictive AST by species compared to Vitek 2, for samples with high quality predictions^*a*^

Species	*N*	Categorical agreement (%)	Major error (%)	Very major error (%)
*Staphylococcus aureus*	9	97.2	3.2	−
*Escherichia coli*	6	87.8	9.2	23.5
*Klebsiella pneumoniae*	3	93.8	6.5	0
*Pseudomonas aeruginosa*	3	52.4	47.6	−
*Enterococcus faecalis*	1	100	0	−
*Klebsiella aerogenes*	1	83.3	20	0
*Streptococcus pneumoniae*	1	66.7	33.3	−
Overall	24	89.3	10.5	12.1

^*a*^ Note: blank cells reflect no data for calculations (e.g., no resistance seen in that species by reference method).

**TABLE 4:** Performance of predictive AST compared to Vitek 2, by species and compound, where high-quality predictions were available^*a*^

Species	Compound	CA (%)	ME (%)	VME (%)
*Enterococcus faecalis*	Gentamicin	100	0	−
	Erythromycin	100	−	0
	Linezolid	100	0	−
	Teicoplanin	100	0	−
	Vancomycin	100	0	−
*Escherichia coli*	Amikacin	83.3	16.7	−
	Amoxicillin + clavulanic acid	100	0	−
	Ampicillin	83.3	33.3	0
	Cefazolin	40	75	0
	Cefepime	100	0	0
	Cefoxitin	83.3	16.7	−
	Ceftazidime	100	0	0
	Ceftriaxone	100	0	0
	Ciprofloxacin	100	0	0
	Gentamicin	100	0	0
	Meropenem	100	0	−
	Sulfamethoxazole + trimethoprim	60	0	66.7
	Tobramycin	83.3	0	100
	Trimethoprim	83.3	0	25
*Klebsiella (Enterobacter) aerogenes*	Amikacin	100	0	−
	Amoxicillin + clavulanic acid	100	−	0
	Cefazolin	100	−	0
	Ceftazidime	0	100	−
	Ceftriaxone	0	100	−
	Ciprofloxacin	100	0	−
	Gentamicin	100	0	−
	Meropenem	100	0	−
	Sulfamethoxazole + trimethoprim	100	0	−
	Ticarcillin + clavulanic acid	100	0	−
	Tobramycin	100	0	−
	Trimethoprim	100	0	−
*Klebsiella pneumoniae*	Amikacin	100	0	−
	Amoxicillin + clavulanic acid	100	0	0
	Cefazolin	66.7	33.3	−
	Ceftazidime	100	0	−
	Ceftriaxone	100	0	−
	Ciprofloxacin	50	50	−
	Gentamicin	100	0	−
	Meropenem	100	0	−
	Sulfamethoxazole + trimethoprim	100	0	−
	Ticarcillin + clavulanic acid	100	0	−
	Tobramycin	100	0	−
	Trimethoprim	100	0	−
*Pseudomonas aeruginosa*	Amikacin	100	0	−
	Cefepime	33.3	66.7	−
	Ceftazidime	0	100	−
	Ciprofloxacin	33.3	66.7	−
	Gentamicin	66.7	33.3	−
	Meropenem	33.3	66.7	−
	Tobramycin	100	0	−
*Staphylococcus aureus*	Benzylpenicillin	100	0	0
	Ciprofloxacin	100	0	−
	Clindamycin	88.9	14.3	0
	Erythromycin	100	0	0
	Fusidic acid	100	0	−
	Gentamicin	100	0	−
	Linezolid	100	0	−
	Mupirocin	88.9	11.1	−
	Rifampicin	88.9	11.1	−
	Teicoplanin	100	0	−
	Tetracycline	100	0	−
	Vancomycin	100	0	−
*Streptococcus pneumoniae*	Benzylpenicillin	0	100	−
	Erythromycin	100	0	−
	Vancomycin	100	0	−
Overall	All agents	89.3	10.5	12.1

^*a*^ Note: blank cells reflect no data for calculations (e.g., no susceptibility/resistance seen in that species by reference method).

For all 470 AST predictions across 42 samples, including both high-quality and exploratory-only results, CA was 87.7%, with 28.4% VME, and 8.3% MEs (Table 5). VMEs were mainly seen with one isolate of *Aeromonas* sp. (100%; false susceptibility for amoxicillin-clavulanate, meropenem, and trimethoprim), *Pseudomonas aeruginosa* (100%; false susceptibility for ticarcillin-clavulanate), *E. coli* (31.6%; false susceptibility for tobramycin, trimethoprim-sulfamethoxazole, and ticarcillin-clavulanate), *Ochrobactrum anthropi* (33.3%; false susceptibility for ceftriaxone), *Staphylococcus haemolyticus* (50%; false susceptibility for cephalothin, ciprofloxacin, rifampicin, tetracycline), and *Staphylococcus epidermidis* (20%; false susceptibility for cephalothin, ciprofloxacin, and teicoplanin). MEs were seen in *Pseudomonas aeruginosa* (45.5%; false resistance for cefepime, ceftazidime, ciprofloxacin, meropenem) and *Klebsiella pneumoniae* (11.1%, false resistance for cephazolin, cefoxitin, and ciprofloxacin) (Table S2). Not all of the tested compounds achieved satisfactory performance even when high-quality predictions were achieved, with CA ranging from >95% (for amoxicillin-clavulanate, gentamicin, erythromycin, fusidic acid, ticarcillin-clavulanate, and vancomycin) to as low as 55.6% for cephazolin, with high rates of VMEs for trimethoprim-sulfamethoxazole (66.7%), trimethoprim (25%), and tobramycin (100%) (Table 6). The only agents that would pass acceptance criteria (>95% CA, <3% ME, and <1.5% VME) even when only using high-quality predictions would be amoxicillin-clavulanate, erythromycin, and fusidic acid. While vancomycin and ticarcillin-clavulanate had 100% CA and no MEs, the lack of resistant isolates precluded calculation of the rate of VMEs.

**TABLE 5:** Performance of predictive AST by species compared to Vitek 2, for all samples including those with both high confidence and exploratory-only predictions^*a*^

Species	*N*	Categorical agreement (%)	Major error (%)	Very major error (%)
*Staphylococcus aureus*	9	96.6	2.9	7.7
*Staphylococcus epidermidis*	7	85.5	7.1	35
*Escherichia coli*	6	86.5	8.7	30
*Enterobacter cloacae*	3	92.3	7.4	8.3
*Klebsiella pneumoniae*	3	90	11.1	0
*Pseudomonas aeruginosa*	3	50	45.5	100
*Aeromonas* sp.	1	70	0	100
*Enterococcus faecalis*	1	100	0	0
*Haemophilus influenzae*	1	100	0	−
*Klebsiella aerogenes*	1	93.3	9.1	0
*Ochrobactrum anthropi*	1	87.5	0	33.3
*Staphylococcus capitis*	1	83.3	0	50
*Staphylococcus haemolyticus*	1	67.7	0	57.1
*Staphylococcus lugdunensis*	1	100	0	0
*Streptococcus pneumoniae*	1	75	25	−
*Streptococcus pyogenes*	1	100	0	0
*Streptococcus dysgalactiae*	1	100	0	−
Overall	42	87.7	8.3	28.4

^*a*^ Note: blank cells reflect no data for calculations (e.g., no resistance seen in that species by reference method).

**TABLE 6:** Performance of predictive AST compared to Vitek 2 for all compounds, where high-quality predictions were available^*a*^

Compound	CA (%)	ME (%)	VME (%)
Amikacin	92.3	7.7	−
Amoxicillin + clavulanic acid	100	0	0
Ampicillin	83.3	33.3	0
Benzylpenicillin	90	50	0
Cefazolin	55.6	57.1	0
Cefepime	77.8	25	0
Cefoxitin	83.3	16.7	−
Ceftazidime	69.2	33.3	0
Ceftriaxone	88.9	12.5	0
Ciprofloxacin	85.7	15	0
Clindamycin	88.9	14.3	0
Erythromycin	100	0	0
Fusidic acid	100	0	−
Gentamicin	95.5	4.8	0
Linezolid	100	0	−
Meropenem	83.3	16.7	−
Sulfamethoxazole + trimethoprim	77.8	0	66.7
Ticarcillin + clavulanic acid	100	0	−
Tobramycin	92.3	0	100
Trimethoprim	90	0	25
Vancomycin	100	0	−
Overall	89.3	10.5	12.1

^*a*^ Note: blank cells reflect no data for calculations (e.g., no resistance seen in that species by reference method).

**Fig 3:**
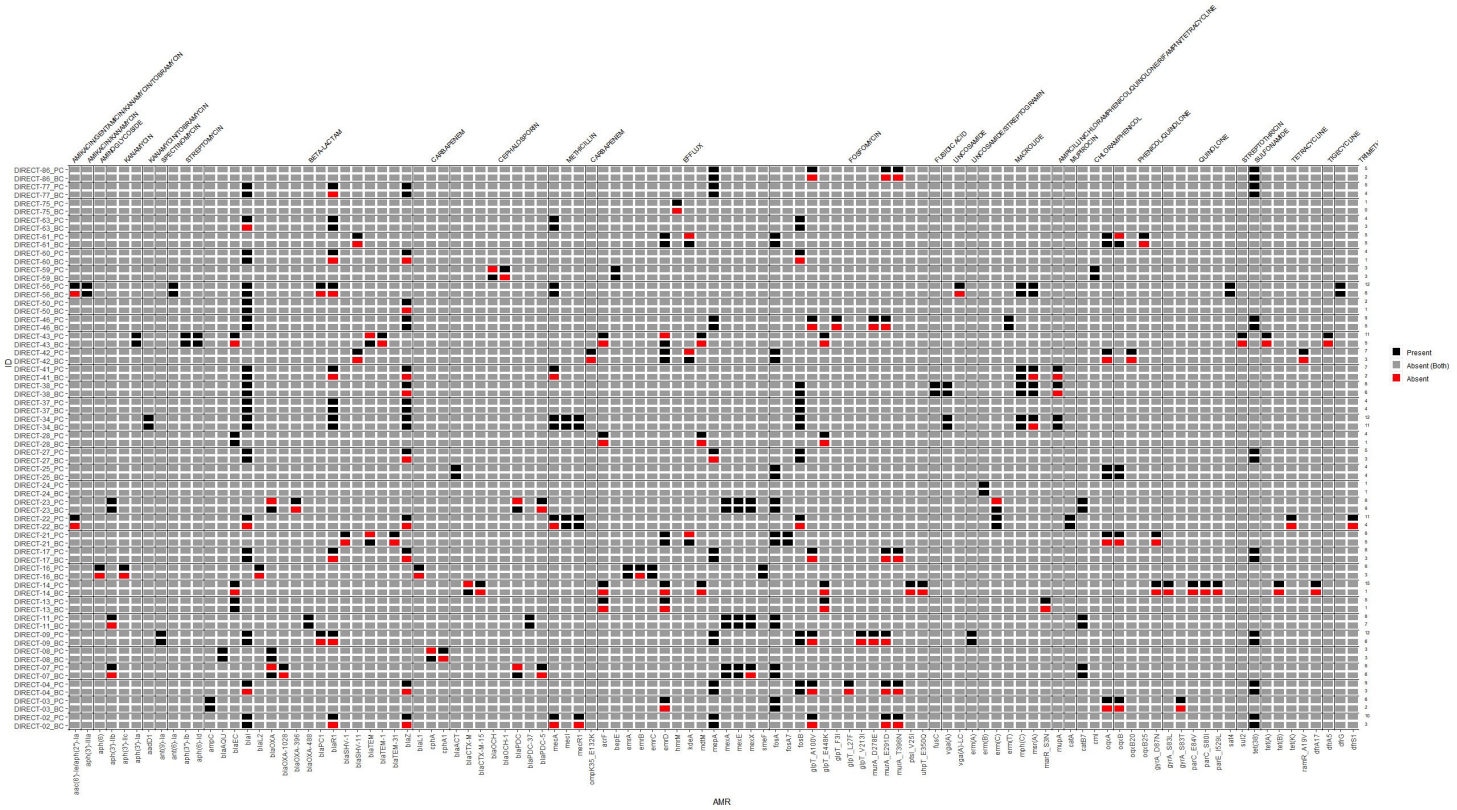
Heat map comparing antimicrobial resistance genes detected from ONT-generated sequences from blood culture broth extracts, compared to Illumina-generated sequences of pure cultured isolates from the same sample. Vertical axis: Sample ID label suffix “PC” = pure culture isolate (sequenced by Illumina); suffix “BC” = blood culture broth (sequenced by ONT).

Detection of AMR genes from ONT-generated assemblies of bacterial genomes from BC broths showed some discrepancies compared to Illumina-based sequencing of pure cultured isolates (Fig. 3) although several of these reflected more specific allele calls from sequencing pure isolates (e.g., *bla*_CTX-M_ from sequencing of BC sample, but *bla*_CTX-M-15_ from pure cultured isolate). Illumina-based sequencing of pure isolates detected a median of 3 additional AMR gene targets (range −1 to 14; IQR 1–5). In only a single sample did ONT detect one more AMR gene target than Illumina.

In terms of potential turn-around times for direct ONT sequencing from blood cultures, pre-sequencing steps took ~4 h, including (a) host DNA reduction, ~2 h; (b) DNA extraction, ~30 min; (c) DNA amplification, ~1 h; and (d) DNA Library prep, ~30 min. Adequate data for downstream processing would usually be achieved within ~4 h, but flow cells were run up to 12 h for single samples and 72 h for multiplexed samples although sequencing beyond 12 h provided limited additional data. The predictive AST calls from AREScloud were available within ~1 h. As such, the potential turn-around time from flagging of a positive blood culture to report generation could be achieved within 9–17 h, potentially faster than conventional AST methods and Illumina-based methods (up to 48 h, or longer).

## DISCUSSION

We describe a ONT-based sequencing approach using positive blood culture broth for pathogen detection and taxonomic classification. In monomicrobial infections, the performance is encouraging, with 100% agreement to genus level. Polymicrobial samples remain challenging, with only one of two pathogens identified in samples encountered in this study. In one polymicrobial sample, identification of *Staphylococcus hominis* only to genus level of *Staphylococcus* could be problematic clinically if prompting clinicians to treat for the more pathogenic *Staphylococcus aureus.* In two samples with species belonging to the *E. cloacae* complex, sequencing-based identification was more accurate than conventional methods, although discrimination by MALDI-TOF of these species is known to be problematic, without additional analysis (25, 26). Sequencing directly from blood samples to detect pathogenic bacteria in patients with sepsis and bloodstream infection is limited by low loads of bacterial DNA in blood at the time of presentation, high concentrations of human DNA, and challenges in discriminating background low-level contaminating DNA (27, 28). Adding a culture-amplification step by sequencing from positive blood culture broths, as described in this study, increases the amount of bacteria DNA available for sequencing.

A key aspiration in the application of genomics-based diagnostics direct from clinical samples would be the ability to accurately predict antimicrobial susceptibility, independently of conventional culture-based methods. However, the presence or absence of resistance genes does not always predict the phenotype, which may be modified by gene expression, gene copy number, and other post-translational effects (29). The machine-learning algorithm used in this study is based on a large sample bank with matched whole-genome sequenced clinical isolates and AST results collected from several international centres (19). Such an approach has advantages in that the algorithm does not require a clear understanding of the association between genotype and phenotype but will learn to use relevant genomic features if supplied with adequate amounts of data.

The use of direct sequencing and predictive AST from positive blood culture broths holds some promise, and ONT long-read sequencing using MinION offers potential time advantages over Illumina-based approaches. Accuracy of pathogen identification was similar to results previously achieved with Illumina (95% species-level agreement) (5). However, using ONT sequencing chemistries, flow cells, and base-callers available at the time of study, AST predictions based on ONT data were considerably less accurate than Illumina-based methods, where we have previously demonstrated CA of >95% for common gram-negative pathogens against 17 antimicrobials (with an overall 11% VME rate) (5). A number of factors are likely to account for performance limitations seen with ONT-based predictive AST in this study, including inadequate genome coverage, database limitations, a relatively small sample size including a limited range of species and resistance phenotypes, loss of plasmids and the use of older flow-cells and kit chemistries available at the time. ONT sequencing in some samples resulted in significant fragmentation and incompleteness of assembled genomes, caused by an insufficient number of reads and low average read length in a subset of data (sequencing metrics in Table S1), as well as lower depth of sequencing compared with Illumina. In addition, reads overall exhibited low per-base accuracy (average Phred score of 10.48, i.e., approximately 90% accuracy), likely due to base-calling with the “fast” profile and the use of an older versions of the Guppy base-caller (v5.0.16; and v3.2.9 used in earlier sequencing runs). Additional work is needed to assess the performance of current ONT consumables (e.g., R10.4.1 flow cells and kit 14 chemistry), which are reported to achieve very high sequencing accuracies (30), and more current base-calling software. More stringent quality thresholds to define adequate genome assemblies may also improve performance. The high rates of MEs/VMEs encountered in common species in this study, would currently preclude application for clinical use. CLSI M52 guidelines recommend that new AST systems demonstrate CA ≥ 90% and rates of MEs and VMEs < 3% (22), although given the high risk of VMEs to patient care, the FDA stipulates VME rates to be <1.5% (31). However, it should be noted that some of these errors occurred in less critical or uncommonly prescribed species/antibiotic combinations (e.g., *E. coli* and tobramycin). It could be hypothesized that errors may be mitigated by database enhancement and training of the algorithms on a larger number and broader range of organisms, but this requires further study. The application of sequencing from blood cultures may also allow faster identification of slow-growing or fastidious organisms. One potential approach to reduce time to reporting might be the early sampling of blood culture broths, before they flag positive on automated detections systems. In this way, there may be adequate pathogen load to undertake sequencing, while reducing the overall turn-around time. However, the relatively low proportion of blood cultures that return bacterial growth may require testing of all inoculated samples, limiting practical application and cost-effectiveness. The cost of ONT sequencing from positive blood cultures is dependent on a number of variables, such as whether single or multiplexed samples are run, whether samples are batched (which reduces costs but may prolong turn-around times) or run “on-demand” or whether flow cells are reused. However, using the extraction methods, library preparation, and sequencing protocols described in this study, and if 10 samples are multiplexed per flow cell, cost estimates range from US$45–65 per sample. One further advantage of ONT long-read sequencing is the ability to sequence plasmid genomes and place AMR genes into context with associated mobile genetic elements. While we did not systematically examine plasmid content in this study, we have previously shown that the DNA extraction methods we used can preserve plasmid structures for ONT sequencing (5).

While there are several rapid molecular diagnostic assays available for species identification and AMR gene detection in clinical use (e.g., BCID2, ePlex, VERIGENE, T2MR, etc.), these usually have relatively limited number of species/AMR gene targets on the panel. A key advantage of a sequencing-based approach is not only the ability to potentially detect any bacterial species in the sample but also to provide an AST output familiar to treating clinicians, i.e., a predictive phenotype of multiple antibiotics to select appropriate therapy, maximizing clinical utility.

Limitations to this study are acknowledged. While samples included in the study were prospectively collected, and included most common species causing sepsis, a more extensive range of pathogens, including diverse AMR phenotypes, would need to be assessed to understand the reliability, broader applicability, and clinical utility of this approach. Furthermore, our collection included few samples with resistance to some agents, inflating VME rate in some species/antibiotic combinations. For example, there were only two *Pseudomonas aeruginosa* isolates with resistance to any antimicrobial tested; with resistance to ticarcillin-clavulanate that were both falsely reported as susceptible by predictive AST, leading to a 100% VME rate (albeit only from low-quality exploratory predictions). The absence of any resistances in the *Pseudomonas* samples to other agents precluded the ability to calculate any VMEs for other antibiotics using high-quality predictions. It is also acknowledged that Vitek 2 AST is not a reference method for MIC determination (such as broth microdilution or agar dilution) and is an imperfect standard for comparison despite being commonly used in clinical diagnostic laboratories. Additionally, improvements in ONT flow cell technology have occurred since this study was performed, including the possibility of adaptive sequencing that can actively exclude human DNA during the sequencing process (32), which may also further improve the application of these methods.

### Conclusions

Direct sequencing from positive blood culture broths in patients with sepsis is feasible and can provide accurate species-level identification of causative pathogens, especially in monomicrobial infections. Predictive AST shows promise for some bacterial species and antibiotic combinations but is sub-optimal for a number of common pathogens with unacceptably high VME rates driven by less reliable calls for certain species and drug combinations. Diagnostic performance characteristics were improved by only accepting results where high-quality predictions were available, compared to all predictions regardless of quality (CA 89.3% vs 87.7%), most notably in reduced rates of VMEs (12.1% vs 28.4%). ONT-based approaches may be faster and provide data in real time, but improvements in accuracy across a broader range of organisms are required before it can be considered for clinical use. Improved performance should be achievable with training of ML algorithms on larger and more diverse data sets, and masking of results where poor performance of certain species and drug combinations are recognized. Furthermore, ongoing developments in the accuracy of rapid sequencing technologies should lead to improved performance of these methods for eventual diagnostic use. The prospect of rapid whole-genome sequencing from culture-amplified clinical samples holds promise and could bring us closer to clinical application for the management of patients with sepsis and bloodstream infection.

## Data Availability

Genome data have been deposited to NCBI under Bioproject PRJNA982891. ONT sequence data from BC broth extracts have been uploaded to the sequence read archive (SRA) under accession numbers SAMN35766863 to SAMN35766914. Illumina sequence reads from pure cultured isolates have been uploaded using accession numbers SAMN35767221 to SAMN35767269. Taxonomic classifications for blood culture samples can be visualized here: https://fordegenomics.github.io/direct

## SUPPLEMENTAL MATERIAL

Supplementary Table S1 spectrum.03065-23-s0001.xlsx Sample meta-data and results.

Supplementary Table S2 spectrum.03065-23-s0002.xlsx AREScloud output by taxon and compound, for exploratory and high quality predictions.

ASM does not own the copyrights to Supplemental Material that may be linked to, or accessed through, an article. The authors have granted ASM a non-exclusive, world-wide license to publish the Supplemental Material files. Please contact the corresponding author directly for reuse.
